# Epileptogenic zone detection in MRI negative epilepsy using adaptive thresholding of arterial spin labeling data

**DOI:** 10.1038/s41598-021-89774-4

**Published:** 2021-05-25

**Authors:** Martin Gajdoš, Pavel Říha, Martin Kojan, Irena Doležalová, Henk J. M. M. Mutsaerts, Jan Petr, Ivan Rektor

**Affiliations:** 1 CEITEC – Central European Institute of Technology, Neuroscience Center, Masaryk University, Brno, Czech Republic; 2 Department of Neurology, Brno Epilepsy Center, St. Anne’s University Hospital and Medical Faculty of Masaryk University, Pekařská 53, Brno, 656 91 Czech Republic; 3 Department of Radiology and Nuclear Medicine, Amsterdam University Medical Center, VU University Medical Center, Amsterdam Neuroscience, Amsterdam, The Netherlands; 4 Department of Radiology and Nuclear Medicine, University Hospital Ghent, Ghent, Belgium; 5 Institute of Radiopharmaceutical Cancer Research, Helmholtz-Zentrum Dresden-Rossendorf, Dresden, Germany

**Keywords:** Neuroscience, Medical research, Neurology

## Abstract

Drug-resistant epilepsy is a diagnostic and therapeutic challenge, mainly in patients with negative MRI findings. State-of-the-art imaging methods complement standard epilepsy protocols with new information and help epileptologists to increase the reliability of their decisions. In this study, we investigate whether arterial spin labeling (ASL) perfusion MRI can help localize the epileptogenic zone (EZ). To that end, we developed an image processing method to detect the EZ as an area with hypoperfusion relative to the contralateral unaffected side, using subject-specific thresholding of the asymmetry index in ASL images. We demonstrated three thresholding criteria (termed minimal product criterion, minimal distance criterion, and elbow criterion) on 29 patients with MRI-negative epilepsy (age 32.98 ± 10.4 years). The minimal product criterion showed optimal results in terms of positive predictive value (mean 0.12 in postoperative group and 0.22 in preoperative group) and true positive rate (mean 0.71 in postoperative group and 1.82 in preoperative group). Additionally, we found high accuracy in determining the EZ side (mean 0.86 in postoperative group and 0.73 in preoperative group out of 1.00). ASL can be easily incorporated into the standard presurgical MR protocol, and it provides an additional benefit in EZ localization.

## Introduction

Epilepsy is a neurological disease with a prevalence of about 1%. About one third of the patients diagnosed with epilepsy do not respond to standard antiepileptic medication^1^. Epilepsy surgery is optimal in these drug-resistant patients. Unfortunately, no identifiable epileptogenic lesion is found in 20% to 40% of epilepsy surgery candidates using the anatomical magnetic resonance imaging (MRI) that is part of the standard presurgical MRI protocol^2^. Therefore, it is necessary to extend the structural imaging in these cases with other techniques measuring the physiology and pathophysiology of the brain.

The localization of the epileptogenic zone (EZ) is currently based on the convergence of clinical results, interictal/ictal EEG, and imaging techniques. Regional brain perfusion is one of the physiological parameters that may contribute to the EZ localization, particularly in MRI-negative epilepsy^3^.

Alterations in brain physiology can be measured by interictal and ictal single-photon emission tomography (SPECT) and their subtraction (subtraction ictal SPECT co-registered to MRI; SISCOM), and by fluorodeoxyglucose positron emission tomography (FDG-PET). However, these methods have their limitations, including the use of radiotracers, higher costs, and the necessity of injecting the tracer at the very beginning of the seizure in SISCOM imaging. An alternative method for imaging perfusion is arterial spin labeling (ASL) MRI. ASL is generally accessible as a product sequence on most MRI machines, is completely non-invasive, and has clinically feasible scanning times. Recent progress in the standardization of ASL acquisition^4^, data sharing, and processing^5^ made this a common tool for clinical research with increasing adoption into clinical practice^6^. ASL was demonstrated to be helpful in several types of epilepsy, including temporal lobe epilepsy^7,8^ and frontal lobe epilepsy^8,9^. One major remaining hurdle to the application of ASL for detecting EZ is the definition of EZ on ASL perfusion images in a robust way, especially considering the physiological variability between patients^10^ and inter-scanner quantification challenges^5^.

In this study, we focused on EZ detection in MRI-negative epilepsy based on ASL data. ASL has been proven to be useful in EZ localization in this type of epilepsy^3,10^. ASL plays an important role in EZ localization in combination with other methods^11^. In this work, we focus on processing ASL independently of other methods suitable for EZ detection; we intend to present ASL as one of the methods important for reaching a final decision on EZ localization in MRI-negative epilepsy.

The main problem associated with ASL is the strong inter-individual variability in perfusion data. Some studies attempted to overcome this issue using variability modeling^10^. Subject-specific thresholding might also be beneficial. We tried to solve this problem using subject data as control data, thus completely avoiding the problem of interindividual variability by not comparing patient data with variable groups of healthy controls.

The asymmetry index (AI) is a suitable approach for analyses of metabolic or perfusion data independent of the selection and size of the regions of interest. It is based on the hemispheric asymmetries of a measured parameter and is often used in studies focused on ASL data in epileptic patients^3,7,12–17^. Perfusion-based approaches for EZ localization can exploit known interictal hypoperfusion caused by changes of perfusion brain dynamics in the seizure onset zone^18^. In many studies, the analysis of AI is based on statistical comparisons (e.g. t test) with healthy controls^7,14,16,17^ and often use a ROI-based approach^7,16,17^. An alternative to comparison with healthy controls is to rely on patient data-specific thresholding of AI. Previous approaches to thresholding used AI z values z_AI_ with thresholding |z_AI_|> 1.64 (corresponds to p < 0.05)^3^ or used the 95th percentile as a decisive threshold^12^.

Our aim was to propose a straightforward and simple EZ localization workflow for ASL data that is feasible in a clinical environment and makes it possible to identify the EZ with high robustness. In addition to accurate preprocessing, an important step of the analysis is the optimal thresholding of perfusion data, ensuring both high sensitivity and specificity. To that end, we proposed a method that identifies the EZ using an asymmetry index of cerebral blood flow (CBF) and compared three approaches to obtaining a subject-specific threshold based on an exponential fit to a normalized histogram of the CBF. Our proposed method is independent of the selection of the parcellation scheme. Moreover, acquisition of healthy control data for calibration is not needed. We tested the proposed approach for EZ detection in two groups of epilepsy surgery candidates with MRI-negative epilepsy. First, we tested the proposed method retrospectively in patients who underwent successful resection with satisfying results. Second, we prospectively tested the method in a group of presurgical candidates for whom the hypothesis about EZ was based on a standard presurgical evaluation. We compared our approach with thresholding methods used previously in the literature^3,12^.

## Methods

Our approach was divided into two steps. First, we examined three methods for EZ localization in the group that that underwent successful resection with satisfying results (POST group). The ASL results in this group were confirmed by positive histopathological findings and successful surgery. Second, the methodology was prospectively tested in the preoperative group (PRE group) for whom the EZ localization was established based on a clinical hypothesis after completing the standard evaluation protocol.

### Subjects

We evaluated the data of 29 patients with drug-resistant epilepsy who were MRI-negative and underwent a complete presurgical evaluation at the Brno Epilepsy Center between 2017 and 2020. The MRI-negative epilepsy definition was based on a routine 3 T MRI examination according to a presurgical epilepsy protocol evaluated by an experienced neuroradiologist^19^. We included all eligible patients with clinically normal MRI. The patients with visible anomalies were excluded from the study.

The study was approved by the ethics committee of Masaryk University and by the ethics committee of St. Anne’s University Hospital. This study was designed in accordance with the Declaration of Helsinki. All patients gave their informed consent before entering the study.

The standard protocol for presurgical evaluation included the following examination: (1) interictal and ictal EEG, (2) 3 T MRI, (3) qualitative analysis of FDG-PET, (4) neuropsychological assessment, and (5) interictal/ictal SPECT and their post-processing SISCOM (SISCOM was performed only in clinically indicated patients).

The patients were divided into two groups: the preoperative group (PRE group) and the postoperative group (POST group).

In the PRE group, there were 22 epilepsy surgery candidates (mean 34 ± 10.2 years, 8 females). In the PRE group, the hypothesis on EZ localization was based on the standard protocol as defined above. We defined 10 possible areas of EZ: the right- or left-sided insula, frontal, temporal, parietal and occipital lobes. The masks were created according to the AAL atlas^20^. ROIs belonged to one of ten defined areas.

In the POST group, there were 7 patients (mean 32 ± 10.3 years, 3 females) who underwent epilepsy surgery with complete or substantial seizure cessation characterized according to the International League Against Epilepsy (ILAE)^21^ as class 1 (5 patients) or class 2 (2 patients). In all patients, invasive EEG was performed as a part of the presurgical evaluation. The EZ localization was confirmed by positive histopathology^22,23^. Based on the resection borders in post-resection MRI, we delineated the resection mask for each patient and approximated it for calculations as the mask of EZ.

### Data acquisition

MRI data were acquired with a 3.0 T MAGNETOM Siemens Prisma. Perfusion imaging was performed using pseudo-continuous ASL (PCASL) with 2D EPI readout without background suppression^24^. We acquired 41 pairs of control and label images; each image consisted of 21 axial slices with 0.6 mm gap between slices, slice thickness = 6 mm, repetition time (TR) = 4079 ms, echo time (TE) = 16 ms, FOV = 192 × 192 mm, flip angle (FA) = 90°, matrix size = 64 × 64, bandwidth = 2056 Hz/Px, echo train length = 48, post labeling delay (PLD) = 1800 ms, labeling duration (LD) = 1800 ms, and slice readout time 30 ms. For quantification calibration, we acquired an M0 scan with TR = 8000 ms. All perfusion data were acquired in 355 s.

The MRI acquisition followed with a high-resolution anatomical T1-weighted image using the MPRAGE sequence with 240 sagittal slices, TR = 2300 ms, TE = 2.34 ms, FOV = 256 × 260 mm, flip angle = 8°, matrix size 256 × 260, slice thickness = 1 mm, bandwidth = 190 Hz/Px.

### Data preprocessing

Preprocessing of ASL data was performed with the ExploreASL pipeline^5^ and consisted of motion correction, exclusion of motion outliers using the ENABLE algorithm^25^, CBF quantification according to the consensus paper^4^, and partial volume correction by linear regression^26^.

We calculated a voxelwise between-hemisphere asymmetry index (AI)) of the CBF maps in voxel × ^13^:

$$ {\text{AI}}_{{\text{x}}} = { 1}00*\left( {{\text{CBF}}_{{\text{x}}} {-}{\text{ CBF}}_{{\text{y}}} } \right) \, / \, \left( {{\text{CBF}}_{{\text{x}}} + {\text{ CBF}}_{{\text{y}}} } \right) $$

Here *y* is position of the corresponding voxel in the contralateral hemisphere. We used only negative AI values, which represent interictal hypoperfusion. Finally, AI values were spatially smoothed with 8 mm FWHM. The AI map was calculated only within the a priori mask of intracranial volume.

### Proposed thresholding methods

The EZ is localized by thresholding the AI maps. To achieve an optimal performance of the method in terms of sensitivity and specificity, we proposed and compared three robust methods for subject-specific adaptive thresholding.

For all three methods, we computed a subject-specific Al histogram, normalized its count (nC) and AI range (nAI) axis in a range from 0 to 1, and fitted the exponential curve *f*. For details of AI histograms and curves of exponential fit, see Figures S7 and S8 in the Supplementary information. All proposed thresholding methods for adaptive thresholding of AI are based on parameters of *f*; particularly, the threshold is based on:

1. The minimal distance to point of origin [0,0] (minimal distance criterion);
2. The minimal product of normalized nC and nAI (minimal product criterion);
3. The elbow criterion, i.e. the point at which *f* bends. Briefly, *f* bends at the point where the gradient of its tangent line is equal to -1, which represents a -45 degree slope of the line.

**Figure 1:**
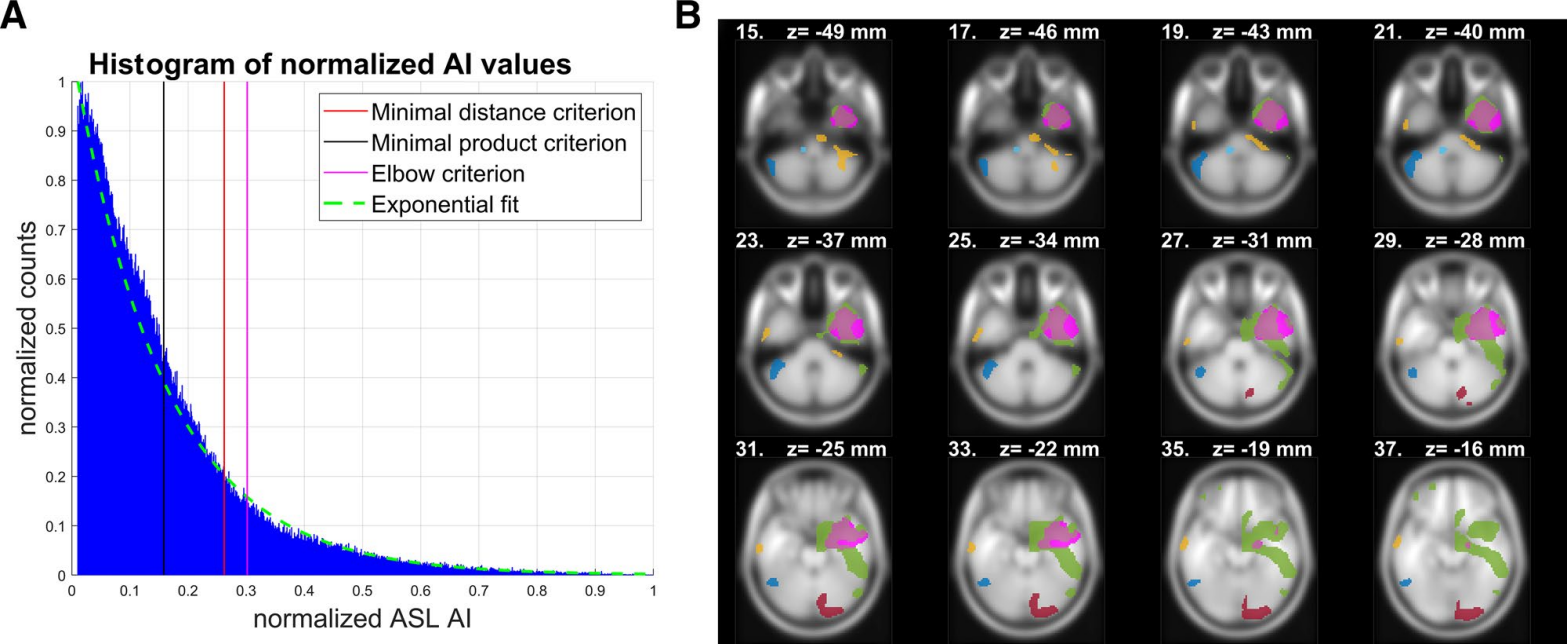
Example of AI (A) histogram of a representative subject from the POST group. Adaptive thresholds are depicted, based on the three proposed thresholding methods. On the right (B) is a slice view with a resection mask (magenta) and clusters (other colors) of AI after thresholding with the minimal product criterion. This was revealed to be the optimal criterion. Used abbreviations: AI—asymmetry index, ASL—arterial spin labeling.

An example of the thresholds and resulting clusters based on the proposed criteria is shown in Fig. 1.

### Metrics for evaluation of accuracy

After thresholding the AI images, we only kept the connected clusters with more than 500 voxels (~ 1.7 ml) for further analyses. This threshold represents an approximate trade-off between the number of involved clusters and the minimal cluster size, as shown in Figure S5 in the Supplementary information. To evaluate the accuracy, we used the following metrics:

(A) Mask hit; a binary criterion that is true only if at least one cluster covers more than 10% of the resection mask (or more than 1% of the EZ hypothesis mask);
(B) Percent of overlay of the resection mask or the EZ hypothesis mask by the most precise cluster;
(C) Precision in determining the EZ side. The predicted EZ side is decided according to the greater sum of suprathresholded AI voxels in the left and right hemispheres. The criterion is binary;
(D) True positive (TP). Number of clusters covering more than 10% of the resection mask or more than 1% of the EZ hypothesis mask;
(E) False positive (FP). Number of clusters outside of the resection/EZ hypothesis mask or covering less than 10% of the resection mask or less than 1% of the EZ hypothesis mask;
(F) Positive predictive value (PPV). Computed as TP divided by the sum of TP and FP.

## Results

When comparing the thresholding methods, the minimal product criterion corresponded on average to 17% of the highest AI values. The minimal distance criterion and the elbow criterion are stricter. The minimal distance criterion corresponded on average to 6%, and the elbow criterion corresponded to 4% of the highest AI values.

The best mask hit was achieved with the minimal product criterion threshold with 71% true positive values for the POST group and 100% true values for the PRE group. The minimal distance criterion and the elbow criterion reached lower amounts of the true values. In both groups, TP was the highest for minimal product criterion; the lowest FP was achieved with the elbow criterion. The optimal trade-off was expressed by PPV, which was in all cases optimal for the minimal product criterion. Details on the performances of all of the used metrics are depicted in Table 1. Details on the comparisons with other thresholding methods mentioned in the literature are summarized in Table S1 in the Supplementary information.

**Table 1:** Evaluation of particular metrics in proposed thresholding methods.

Metric / thresholding method	Minimal distance criterion	Minimal product criterion	Elbow criterion
*PRE group*			
Mask hit	0.73 ± 0.46 (1.00)	**1.00 ± 0.00 (1.00)**	0.68 ± 0.48 (1.00)
Percent of overlay *	5.00 ± 4.49 (3.96)	**15.13 ± 9.74 (13.06)**	3.35 ± 3.38 (1.96)
Precision in EZ side	0.68 ± 0.48 (1.00)	**0.73 ± 0.46 (1.00)**	0.68 ± 0.48 (1.00)
TP	1.32 ± 1.04 (1.00)	1.82 ± 0.96 (2.00)	1.00 ± 0.87 (1.00)
FP	5.73 ± 1.64 (6.00)	6.64 ± 2.15 (6.00)	**4.91 ± 1.74 (5.00)**
PPV	0.18 ± 0.14 (0.17)	**0.22 ± 0.11 (0.19)**	0.16 ± 0.14 (0.17)
*POST group*			
Mask hit	0.57 ± 0.53 (1.00)	**0.71 ± 0.49 (1.00)**	0.43 ± 0.53 (0.00)
Percent of overlay *	17.82 ± 20.03 (12.19)	**31.98 ± 22.82 (35.74)**	14.38 ± 18.31 (9.72)
Precision in EZ side	**0.86 ± 0.38 (1.00)**	**0.86 ± 0.38 (1.00)**	**0.86 ± 0.38 (1.00)**
TP	0.57 ± 0.53 (1.00)	0.71 ± 0.49 (1.00)	0.43 ± 0.53 (0.00)
FP	6.57 ± 1.62 (7.00)	6.29 ± 1.89 (6.00)	**4.86 ± 2.04 (4.00)**
PPV	0.07 ± 0.07 (0.11)	**0.12 ± 0.08 (0.14)**	0.07 ± 0.09 (0.00)

The values are mean ± standard deviation (median). The optimal values are marked in bold. In TP itself, there might not be a clear optimal value in all cases. Therefore we did not mark any TP value in bold. Used abbreviations: TP—true positive, FP—false positive, PPV—positive predictive value, EZ—epileptogenic zone, POST—postoperative group, PRE—preoperative group, *percent of overlay of the resection mask/ EZ hypothesis mask by the most precise cluster.

## Discussion

Our data confirm the applicability of ASL in MRI-negative epilepsy. We proposed a data-driven approach for EZ localization that is easily applicable in clinical practice. Because the calculation of the AI requires little computation time, it could be implemented within a radiology viewer, if brain segmentation and registration are available. The main advantages of this approach are that (1) it is independent of a priori hypotheses about the parcellation scheme, such as the ROI-based analysis using the Harvard–Oxford atlas^10^, and (2) it does not require data from healthy controls as a calibration group for the perfusion template. The main disadvantage of methods requiring a perfusion template is the non-transferability of the template. The perfusion template may vary depending on the specific age of the patients and on the ASL sequence type^10^.

The main objective of our paper was to define the optimal way to use CBF for detecting the EZ in MRI-negative epilepsy. This process, as done by AI computation, leads to the suppression of inter-subject variability in more advanced steps than the variability modeling approach. Additionally, the data-driven approach of adaptative thresholding suppresses part of inter-individual variability.

Interestingly, we observed high accuracy when determining the EZ lateralization (right-sided vs. left-sided). In the POST group, all three criteria identified the EZ side with identically high levels of accuracy. This accuracy reached 88% and failed in only one case. In the PRE group, the accuracy decreased to 69%; this level was reached by both the minimal product criterion and the elbow criterion. Although all of the criteria had similar accuracy in EZ lateralization, the choice of an optimal criterion is crucial for EZ localization.

The results of the metrics for evaluating the precision of the thresholding methods were in concordance in both groups. The mask hit metric represents the sensitivity of AI values to designate the EZ resection mask or EZ hypothesis mask. This metric was high for all of the tested thresholding methods, but it reached the best values for the minimal product criterion. Because AI on perfusion maps is not intended as single decisive method for EZ localization and is characterized with high sensitivity, it is crucial to combine AI-based EZ detection with other methods from the standard presurgical clinical protocol. This can reasonably help in refining the final decision of the EZ location in epilepsy surgery candidates.

TP, the analogical metric to mask hit, is also optimal for the minimal product criterion. Interestingly, identical results for the POST group were caused with maximally one cluster emerging to cover at least 10% of the resection mask. In the PRE group, more clusters could meet the criterion and thus the information was not binary. FP is optimal for the elbow criterion, but when considering PPV, the optimal tradeoff between TP and FP, the minimal product criterion was optimal. Based on PPV, accuracy in determining EZ localization was compared with two thresholding methods mentioned in the literature^3,12^. The minimal product criterion threshold method outperformed both the 95th percentile and the |z_AI_|> 1.64 thresholding methods. We would like to compare the accuracy of the proposed methods with methods using healthy controls as reference, such as in Boscolo Galazzo 2015^10^, but other studies did not report results in comparable detail. We expect a certain variability in the accuracy of these methods dependent not only on epilepsy subtype, but also on the specificity of the perfusion template to participant age and ASL sequence used. Some ASL studies in epilepsy rely on visual assessment of the qCBF maps. These studies, focused not only on MRI-negative epilepsy, report ASL accuracy in localizing the EZ at 60% to 75%; Kim (2016)^27^ reported 75% accuracy (33/44 patients) and Sierra-Marcos (2016)^9^ reported 60% accuracy (15/25 patients). This is in concordance with our mask hit criterion, which is for minimal product criterion in mean 71% (5/7 patients confirmed with histopathology and positive ILAE outcome).

Although the cluster-size-based threshold for clusters involved in the analysis influences the results, for all proposed thresholds in both groups, the PPV was optimal for minimal product criterion. For details, see Figure S6 in the Supplementary information. We also evaluated the quality of the exponential fit on AI histograms and observed a very good Spearman correlation (median 0.983). Absolute values of CBF are not necessary for AI calculation. The division with an M0 scan, which is part of the CBF calculation, will compensate partly for B1 inhomogeneity. However, we assume that the calculation of AI from perfusion-weighted images will perform similarly well for EZ localization.

Although the minimal product criterion is more benevolent, the number of misplaced clusters was only slightly increased in comparison to other proposed thresholding methods. Moreover, when considering the higher ability of resection mask detection (mask hit), we suggest using the minimal product criterion as the optimal thresholding method of the presented approaches.

A limitation of our approach is the weakness of the AI: the inability to detect symmetrical disruptions in perfusion (e.g. bitemporal epilepsy). For this special case, testing CBF data to the perfusion template based on healthy control data would be more suitable. Another limitation is the number of subjects in the POST group: the epileptologists reached a sufficiently reliable decision on EZ localization only in some of the epilepsy surgery candidates. In contrast to the recommendation of a consensus pape^4^, background suppression (BS) of ASL was not used as it was not available at the scanner. The inclusion of BS in future studies could lead to increased SNR and better results. Currently, the usefulness of ASL AI-based methods on EZ localization was compared with the resection mask of patients after successfully performed epilepsy surgery. Future studies may focus on the precise quantification of the impact of ASL-AI-based EZ localization on final decisions in surgery planning.

To conclude, based on mask hit and PPV, the minimal product criterion is optimal for adaptive thresholding of AI. In this study, we presented a novel method for EZ localization based on perfusion data obtained with ASL sequences. We showed that this method is suitable for use in clinical environments. AI indexes computed from ASL data appear to be helpful in EZ localization in MRI-negative epilepsy, especially in combination with other methods from the standard clinical presurgical protocol^11^.

## Supplementary Information

Supplementary Information.
