# Supplementary material for: Epileptogenic zone detection in MRI negative epilepsy using adaptive thresholding of arterial spin labeling data
Source: Sci Rep. 2021 May 25;11:10904. doi: 10.1038/s41598-021-89774-4 (PMC8149682; doi:10.1038/s41598-021-89774-4)
Supplement: Supplementary file 1 — Supplementary Information. [file 41598_2021_89774_MOESM1_ESM.pdf]

Supplementary information

Title

Epileptogenic zone detection in MRI negative epilepsy using adaptive thresholding of arterial spin labeling data

Martin Gajdoš [1], Pavel Říha [1,2], Martin Kojan[1,2], Irena Doležalová[2], Henk-Jan Mutsaerts [3,4], Jan Petr [5], Ivan Rektor* [1,2]

[1] CEITEC – Central European Institute of Technology, Neuroscience Center, Masaryk University, Brno, Czech Republic

[2] Brno Epilepsy Center, Department of Neurology, St. Anne's University Hospital and Medical Faculty of Masaryk University, Brno, Czech Republic

[3] Department of Radiology and Nuclear Medicine, Amsterdam University Medical Center, VU University Medical Center, Amsterdam Neuroscience, Amsterdam, the Netherlands

[4] Department of Radiology and Nuclear Medicine, University Hospital Ghent, Ghent, Belgium

[5] Helmholtz-Zentrum Dresden-Rossendorf, Institute of Radiopharmaceutical Cancer Research, Dresden, Germany

*** Corresponding Author:**
Ivan Rektor
Brno Epilepsy Center
Department of Neurology
St. Anne's University Hospital
Pekařská 53 Brno 656 91
Czech Republic
e-mail: irektor@med.muni.cz
Tel: +420 603 864 046

1.1 Clinical sequences

List of clinical sequences used by radiologists to evaluate the lesionality or non-lesionality of a patient's epilepsy and to determine the hypothesis on epileptogenic zone localization (MAGNETOM Siemens Prisma 3T):

- **T2 TSE transversal** – TR 6100 ms, TE 105 ms, voxel size 0.49x0.49x3 mm, FA 150°
- **T2 FL2D transversal hemo** – TR 852 ms, TE 19.9 ms, voxel size 0.69x0x69x3.9 mm, FA 20°
- **T1 TIR transversal** – 2500 ms, TE 11 ms, voxel size 0.69x0.69x3.3, FA 150°
- **T2 TIRM transversal dark-fluid** – TR 5390 ms, TE 81 ms, voxel size 0.69x0.69x3.6 mm, FA 150°
- **T1 TIR coronal p2** – TR 2900 ms, TE 10 ms, voxel size 0.5x0.5x2.2 mm, FA 150°
- **T2 TIRM coronal dark-fluid** – TR 9000 ms, TE 81 ms, voxel size 0.69x0.69x3.6 mm, FA 150°
- **T2 TSE coronal 448 2mm hippocampus** – TR 8000 ms, TE 52 ms, voxel size 0.39x0.39x2 mm, FA 150°
- **T1 MPRAGE sagittal p2 iso** – TR 2300 ms, 2.33 ms, TI 900 ms, voxel size 1x1x1 mm, FA 8°
- **T1 MP2RAGE sagittal p2 iso** – TR 5000 ms, TE 2.97 ms, TI1 766 ms, TI2 2500 ms, voxel size 1x1x1 mm, FA1 4°, FA2 5°
- **T2 SPC FLAIR sagittal p2 iso** – TR 6000 ms, TE 387 ms, TI1 1900 ms, voxel size 1x1x1x mm

1.2 Figures

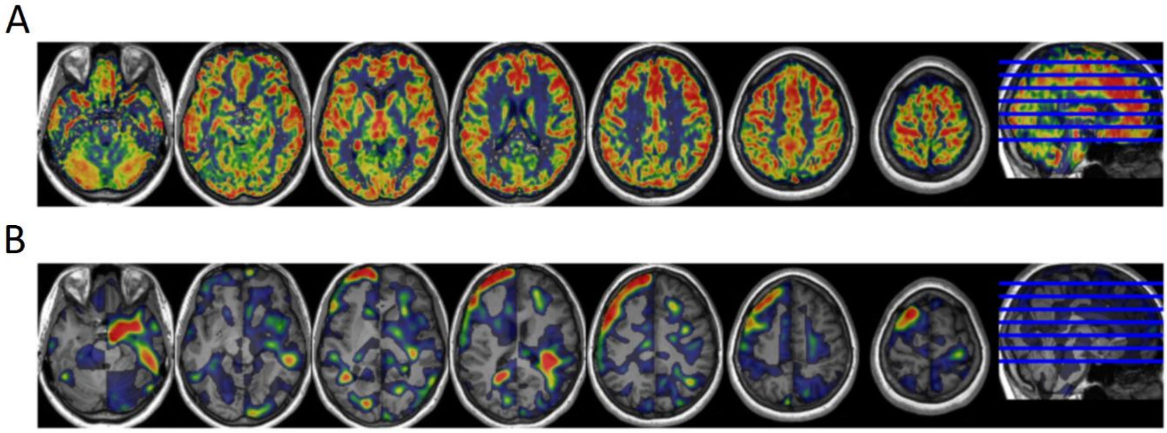

Figure S1. Example of CBF (A) and AI (B) images as transaxial slices overlaid on an anatomical high-resolution image of a representative subject from the POST group. Used abbreviations: CBF – cerebral blood flow, AI – asymmetry index, POST – postoperative group.

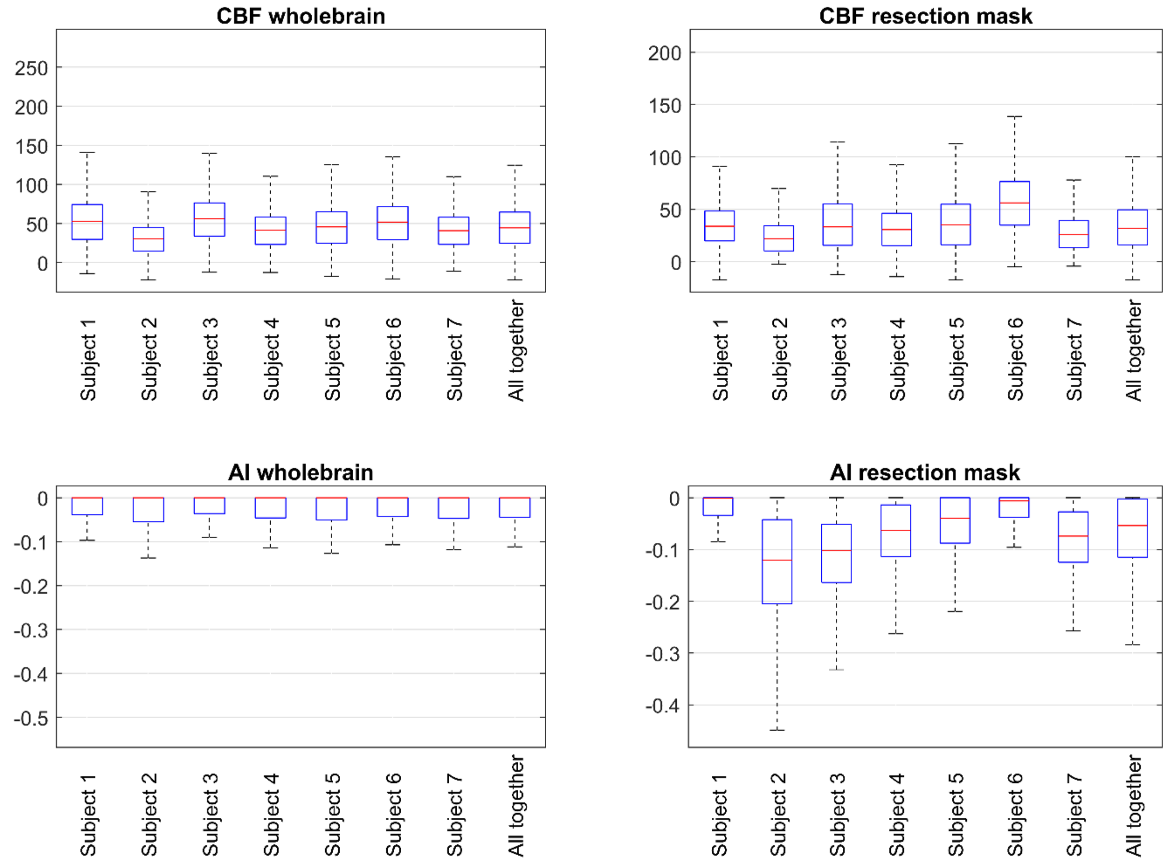

Figure S2. Histograms of CBF and AI values in a whole-brain (left) and a resection mask (right). The values are depicted for all subjects from the POST group. The last box in each boxplot represents all values together. The red line in the box represents median, the blue box represents the first and third quartiles, the dotted line represents the range between a minimal and a maximal value. Outlying values are marked with a red cross. Used abbreviations: CBF – cerebral blood flow, AI – asymmetry index, POST – postoperative group.

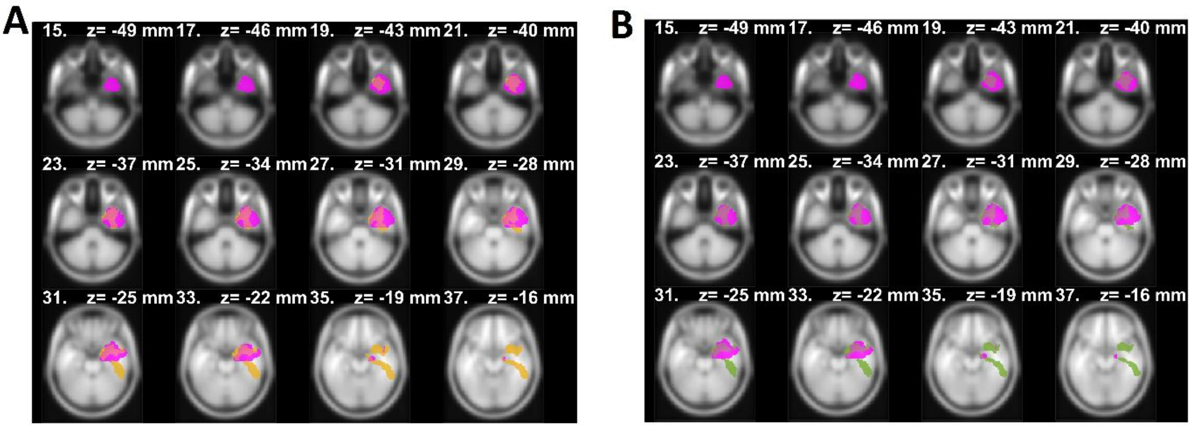

Figure S3. Slice view with a resection mask (magenta) and clusters (other colors) of AI after thresholding with the minimal distance criterion (A) and elbow criterion (B). In this subject, using the minimal distance criterion / minimal product criterion (depicted in Figure 1) / elbow criterion were 44.01% / 56.64% / 38.59% of the resection mask overlaid by the most precise cluster. Used abbreviations: AI – asymmetry index.

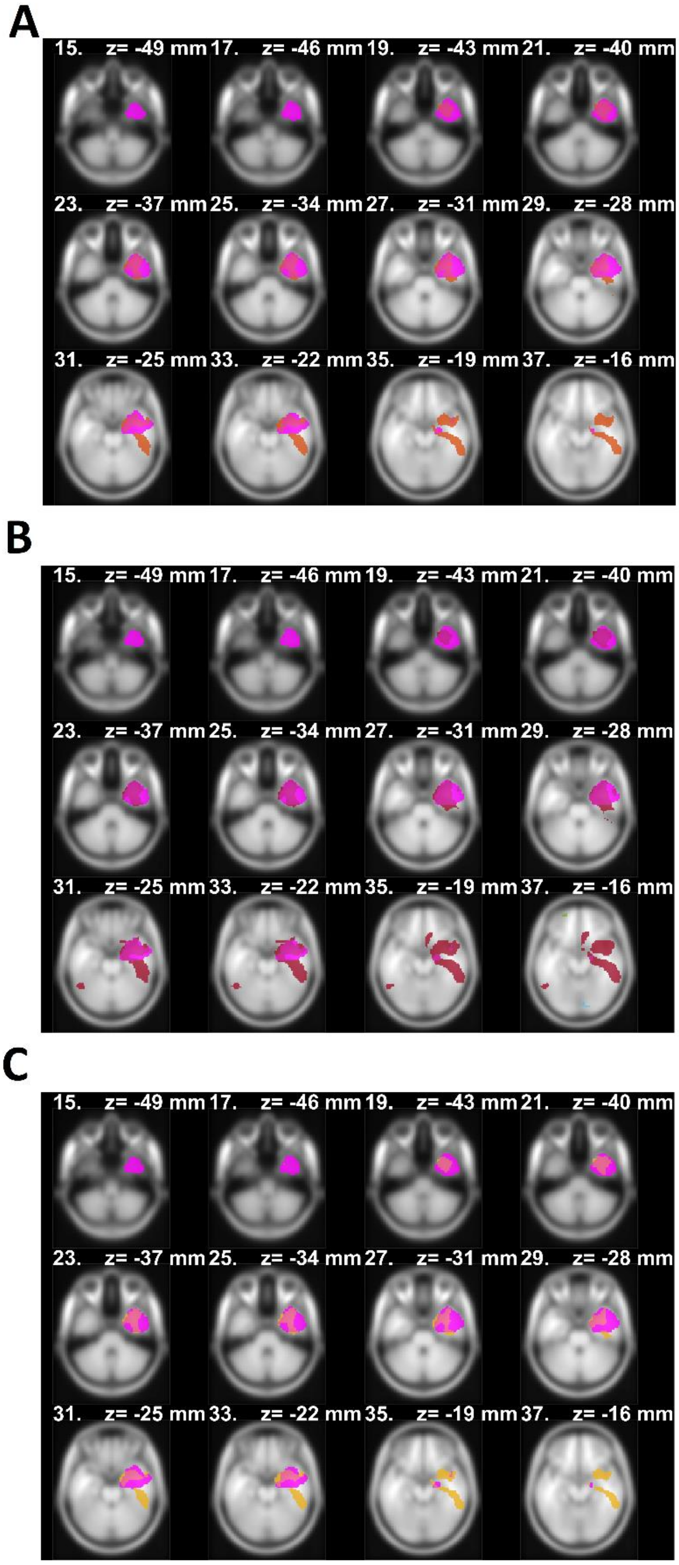

Figure S4. Slice view with a resection mask (magenta) and clusters (other colors) of AI after thresholding with the minimal distance criterion (A), minimal product criterion (B), and elbow criterion (C). Here we present the same subject, but we took into account all clusters with more than 250 vx (instead of 500 vx as presented in the paper). Used abbreviations: AI – asymmetry index.

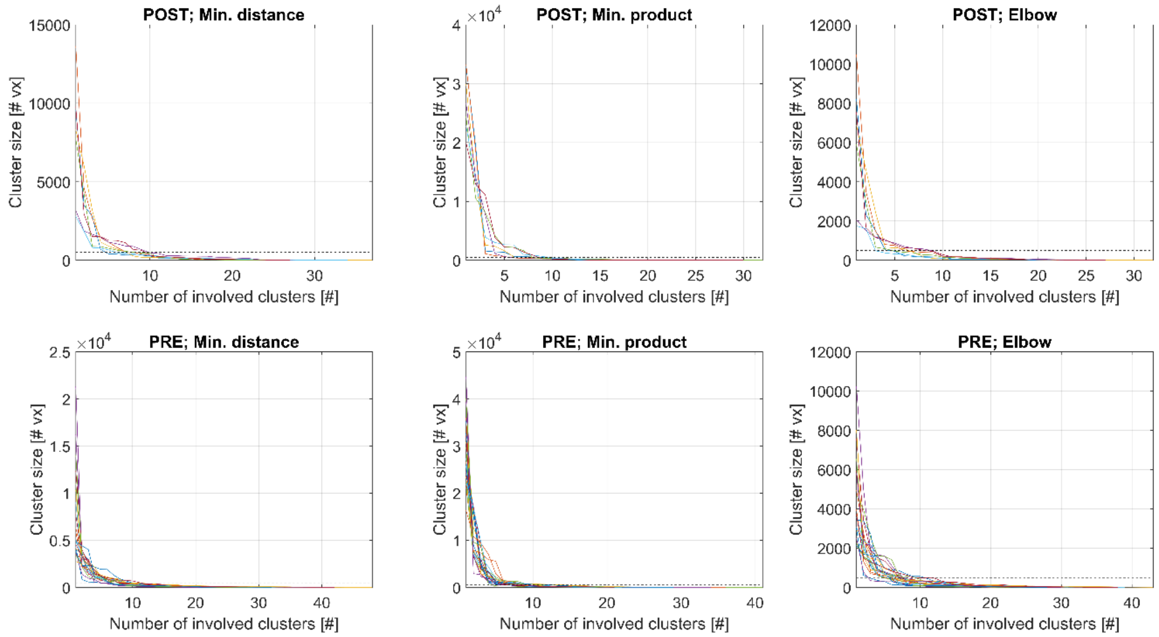

Figure S5. Dependence of cluster size on number of involved clusters larger than 5vx. Clusters are sorted in descending order based on their size. Graph represent each subject in the groups POST (top) and PRE (bottom) and for particular criteria; here minimal distance criterion (left), minimal product criterion (middle), and elbow criterion (right). The threshold of 500 voxels is marked with a dotted line. The selected threshold of 500 voxels for involved clusters is approximately the point where presented dependency bends. Used abbreviations: POST – postoperative group, PRE – preoperative group, vx – voxel.

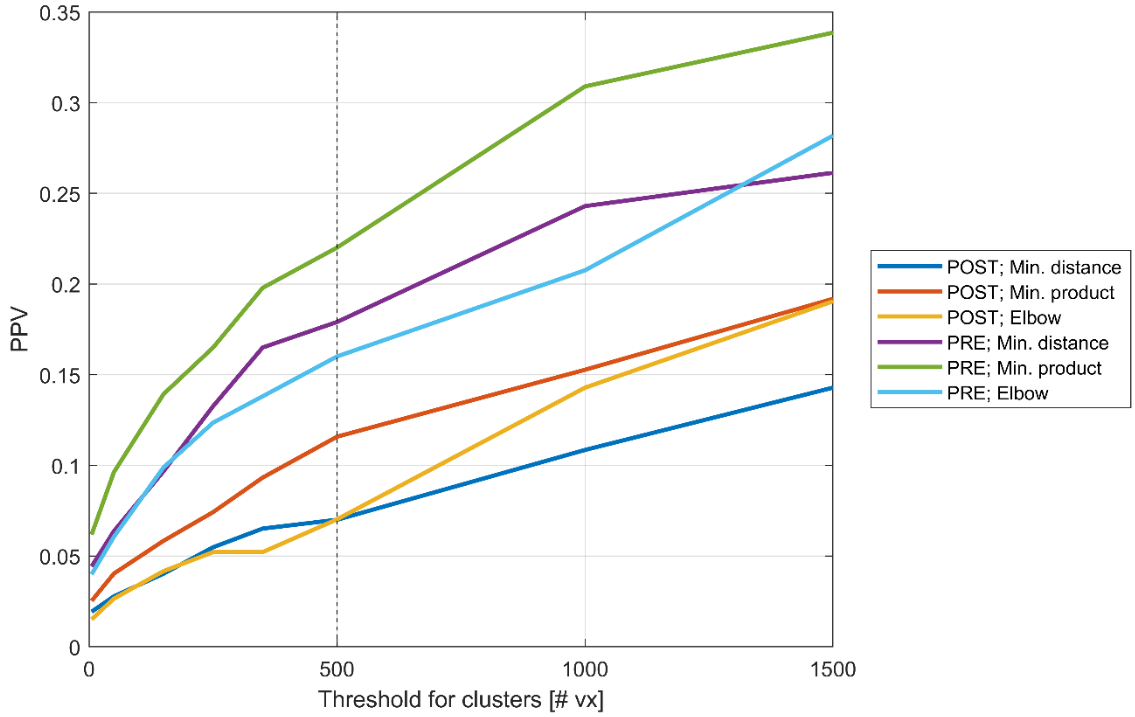

Figure S6. Dependence of PPV on voxel threshold for clusters involved in the analysis. PPV are mean values across the groups POST or PRE for minimal distance, minimal product and elbow criteria. The 500-voxel threshold is marked with a dotted line. PPV values were computed for thresholds [5, 50, 150, 250, 350, 500, 1000, 1500] of voxels. PPV, the optimal trade-off between TP and FP, is highest for minimal product criterion in all the observed thresholds. Used abbreviations: POST – postoperative group, PRE – preoperative group, vx – voxel, PPV – positive predictive value, TP – true positive, FP – false positive.

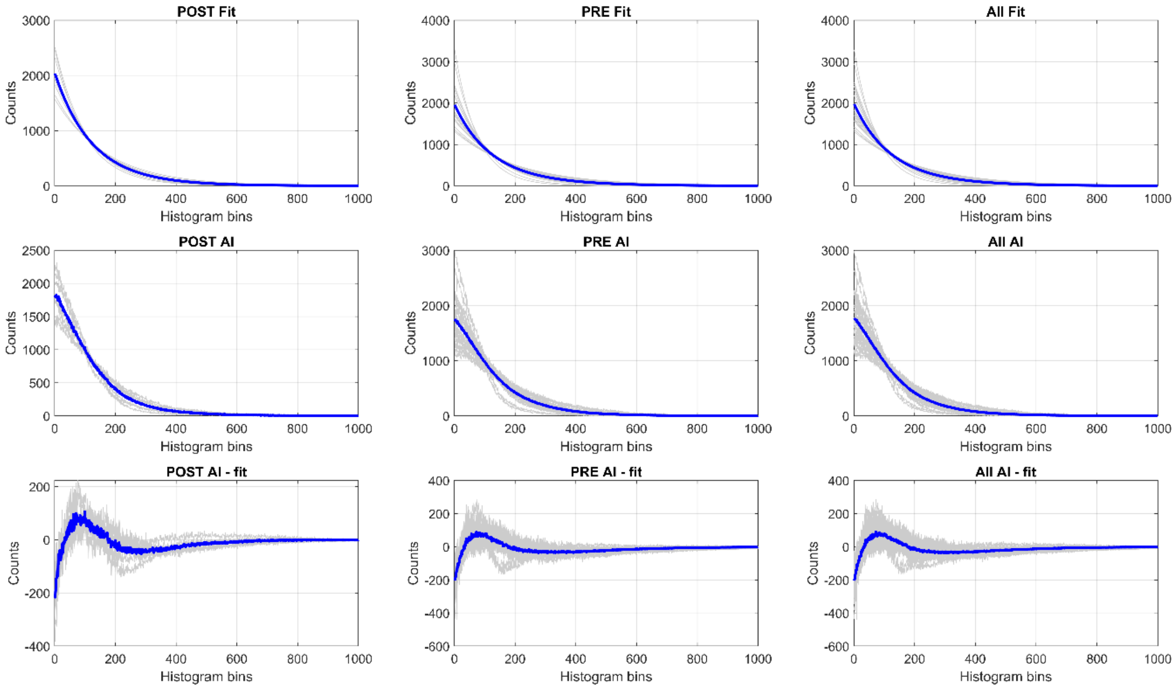

Figure S7. Graphs of fitted exponential curves (top), AI histograms sorted into 1000 bins (middle), and differences between fit and AI histogram data (bottom). Curves are depicted in gray for each subject of the groups POST (left), PRE (middle), and all together (right). The blue curve represents mean across subjects. Used abbreviations: POST – postoperative group, PRE – preoperative group, AI – asymmetry index.

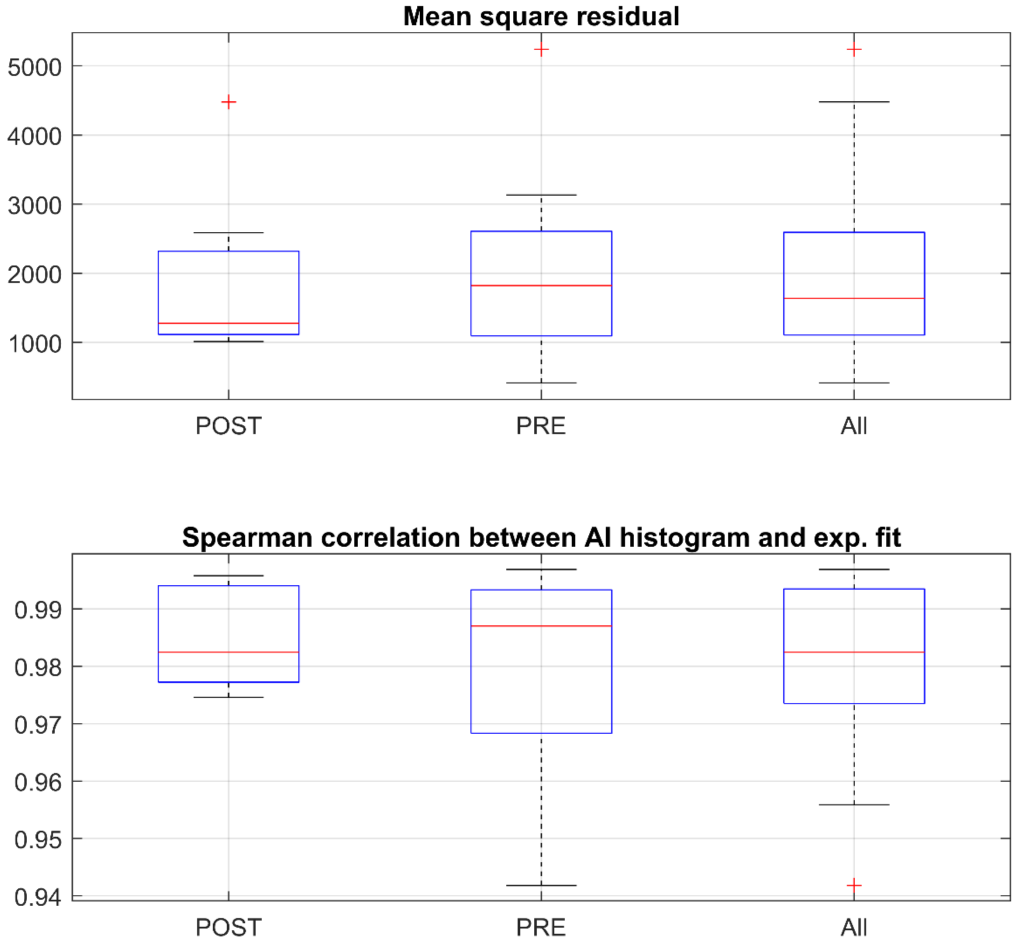

Figure S8. Boxplots of mean square residuals (top) for presenting goodness of exponential fit to histograms of AI. Boxplots represent data from the groups POST, PRE, and all data together. Spearman correlation coefficients between AI histograms and exponential fit curves (bottom) are also presented in boxplots for each of the mentioned groups. In each boxplot, the red line represents the median, the blue box the first and third quartile, and the black line is for the range between minimum and maximum. Outliers are marked with a red cross. Used abbreviations: POST – postoperative group, PRE – preoperative group, AI – asymmetry index.

1.3 Tables

PRE group					
Metric / thresholding method	95 th percentile	|z AI|>1.64	Minimal distance criterion	Minimal product criterion	Elbow criterion
Mask hit	0.77 ± 0.43 (1.00)	0.73 ± 0.46 (1.00)	**1.00 ± 0.00 (1.00)**	0.68 ± 0.48 (1.00)	0.77 ± 0.43 (1.00)
Percent of overlay *	3.72 ± 3.20 (2.48)	5.00 ± 4.49 (3.96)	**15.13 ± 9.74 (13.06)**	3.35 ± 3.38 (1.96)	3.72 ± 3.20 (2.48)
Precision in EZ side	0.68 ± 0.48 (1.00)	0.68 ± 0.48 (1.00)	**0.73 ± 0.46 (1.00)**	0.68 ± 0.48 (1.00)	0.68 ± 0.48 (1.00)
TP	1.18 ± 0.85 (1.00)	1.32 ± 1.04 (1.00)	**1.82 ± 0.96 (2.00)**	1.00 ± 0.87 (1.00)	1.18 ± 0.85 (1.00)
FP	5.86 ± 1.96 (5.50)	5.73 ± 1.64 (6.00)	6.64 ± 2.15 (6.00)	**4.91 ± 1.74 (5.00)**	5.86 ± 1.96 (5.50)
PPV	0.18 ± 0.13 (0.17)	0.18 ± 0.14 (0.17)	**0.22 ± 0.11 (0.19)**	0.16 ± 0.14 (0.17)	0.18 ± 0.13 (0.17)
POST group					
Metric / thresholding method	95 th percentile	|z AI|>1.64	Minimal distance criterion	Minimal product criterion	Elbow criterion
Mask hit	0.43 ± 0.53 (0.00)	0.43 ± 0.53 (0.00)	0.57 ± 0.53 (1.00)	**0.71 ± 0.49 (1.00)**	0.43 ± 0.53 (0.00)
Percent of overlay *	14.75 ± 16.47 (9.99)	14.22 ± 15.90 (9.91)	17.82 ± 20.03 (12.19)	**31.98 ± 22.82 (35.74)**	14.38 ± 18.31 (9.72)
Precision in EZ side	0.86 ± 0.38 (1.00)	0.86 ± 0.38 (1.00)	0.86 ± 0.38 (1.00)	**0.86 ± 0.38 (1.00)**	0.86 ± 0.38 (1.00)
TP	0.43 ± 0.53 (0.00)	0.43 ± 0.53 (0.00)	0.57 ± 0.53 (1.00)	**0.71 ± 0.49 (1.00)**	0.43 ± 0.53 (0.00)
FP	6.57 ± 1.90 (7.00)	5.57 ± 2.51 (6.00)	6.57 ± 1.62 (7.00)	6.29 ± 1.89 (6.00)	**4.86 ± 2.04 (4.00)**
PPV	0.07 ± 0.09 (0.00)	0.09 ± 0.12 (0.00)	0.07 ± 0.07 (0.11)	**0.12 ± 0.08 (0.14)**	0.07 ± 0.09 (0.00)

Table S1. Evaluation of particular metrics in proposed thresholding methods used in Kojan 2021 (95th percentile) and Galazzo 2016 (|z AI|>1.64). The values are mean ± standard deviation (median). The optimal values are marked in bold. Used abbreviations: TP – true positive, FP – false positive, PPV – positive predictive value, EZ – epileptogenic zone, POST – postoperative group, PRE – preoperative group, *percent of overlay of the resection mask/ EZ hypothesis mask by the most precise cluster.
